# Female Entrepreneurial Intentions in Pakistan: A Theory of Planned Behavior Perspective

**DOI:** 10.3389/fpsyg.2021.553963

**Published:** 2021-05-28

**Authors:** Ambreen Sarwar, Qurratulain Ahsan, Nazia Rafiq

**Affiliations:** ^1^Department of Management Sciences, Virtual University of Pakistan, Lahore, Pakistan; ^2^Department of Management Sciences, COMSATS University Islamabad, Lahore Campus, Lahore, Pakistan; ^3^Lahore Business School, The University of Lahore, Lahore, Pakistan

**Keywords:** theory of planned behavior, psychological capital, commitment, intentions, social support, entrepreneurship, Pakistani women

## Abstract

With theoretical underpinnings in the theory of planned behavior, this research aims at investigating how women's entrepreneurial intentions might develop in Pakistan. The survey of 216 female students revealed that psychological capital plays an important role in shaping women's entrepreneurial commitment which in turn results in increased intentions to opt for entrepreneurship as a career. Additionally, it was observed that social support moderates the indirect relation in such a way that in the presence of high social support, the association between psychological capital and intentions *via* commitment is further strengthened. Because women face comparatively more barriers in paid career, therefore it was necessary to study the mechanism and driver that can improve their entrepreneurial intentions, since they represent an untapped resource that might be utilized to improve the economic prospects of a country. The study bridges a significant knowledge gap in utilizing psychological capital to enhance female's entrepreneurial intentions, who are under constant pressure of juggling multiple roles at work and home.

## Introduction

Entrepreneurship means “owning and managing a business on one's own account and risk” (Sternberg and Wennekers, 2005, p. 193). It encompasses activities like recognition of opportunity, organizing, initiating, and administering a business venture (Chell, 2013). Entrepreneurship is amongst the foremost driving features of socioeconomic growth and expansion (Coulibaly et al., 2018). Entrepreneurs play a crucial part in the economic prosperity of a country due to their incredible involvement in economic development (Romer, 1994; Mahfud et al., 2020). Entrepreneurship is considered as a powerful way of economic expansion (Schumpeter, 1934), innovative work opportunities, and sustainable work (Shane and Venkataraman, 2000).

During the last several decades, females have advanced substantially in entrepreneurship and new business development (Kickul et al., 2008); subsequently, women entrepreneurship and related socio-economic growth are of increasing attention. Concomitantly, research into female entrepreneurship has received more focus since the late 1990's (Greer and Greene, 2003; Arenius and Kovalainen, 2006; Langowitz and Minniti, 2007; Ettl and Welter, 2010). However, though female entrepreneurs are increasing gradually in developed countries (Camelo-Ordaz et al., 2016), empirical evidence still suggests that female entrepreneurial ventures are lesser as compared to those owned by male owners and that number of males becoming entrepreneurs are double than women (Gupta et al., 2005). According to the Global Entrepreneurship Monitor (GEM), a project that has scrutinized entrepreneurial activities all around the world, the proportion of females to males with respect to entrepreneurship is less, showing low involvement of females (Minniti et al., 2005; Langowitz and Minniti, 2007; Kwong et al., 2009).

Researchers have demonstrated that intention plays an important part in the decision-making process of initiating a new business venture (Barba-Sánchez and Atienza-Sahuquillo, 2018). Intentions are described as intrinsic motives that drive people to accomplish particular acts (Ajzen, 1991; Lee et al., 2011). Earlier studies have emphasized on the significance of personal attitude, psychological, and social features in the generation of entrepreneurial intentions (EI) (Liñán and Santos, 2007; Ajzen, 2011; Ghani et al., 2013). Nevertheless, until now, there is an inadequate comprehension of the predictors of enhancing EI in female Pakistani students. Particularly, knowledge is limited about the process by which the predicting variables—including psychological factors, attitude, and social factors—enhance entrepreneurial intention (Arshad et al., 2016; Mahfud et al., 2020) in Pakistani females (Sarwar and Imran, 2019).

With the recognition that initiating an entrepreneurship is an sintentional behavior, EI are significant antecedents of planned behavior toward venture development (Bird, 1988; Fayolle et al., 2007). Because intentions act as a proxy for actual actions (Lepoutre et al., 2011; Tausch and Becker, 2013), EI models have tried to recognize the predictions of these intentions (Ajzen, 1991; Krueger et al., 2000).

## Theoretical Background

The theory of planned behavior (TPB; Ajzen, 1991) puts forward that human actions are planned in expectation of the possible outcomes (Ephrem et al., 2019). Consequently, the choice of opting for entrepreneurship is voluntary (Cheng and Liao, 2017). Additionally, the more are the intentions to choose entrepreneurship as a career, the more are the chances that he or she will become an entrepreneur (Contreras et al., 2017a). The intentions arise from an individual's perceived behavioral control (PBC), attitudes, and subjective norm, which are the immediate predictors of intentions. TPB says that the more positive the subjective norms and attitude are, and the better the perceived control, the stronger would be an individual's intention to complete the action. Furthermore, the more is the actual control of the person over the act, he/she is anticipated to translate their intentions into actual action, as soon as opportunity comes (Ajzen, 2006).

Earlier researchers have widely applied the TPB, for shedding light on EI and new venture creation procedure. In this regard, subjective norms (perceived), attitude and PBC have been labeled as antecedents of EI (Ajzen, 2002; Liñán and Fayolle, 2015). The empirical evidence as well as theoretical justifications recommends that attitude (e.g., Goethner et al., 2009), PBC (e.g., Schlaegel and Koenig, 2014), and EI (e.g., Thompson, 2009) be deemed and treated as multidimensional constructs. The current study is encouraged by such earlier researches but takes its point of flight by utilizing a broader approach. It theorizes that EI is driven by psychological capital, through entrepreneurial commitment, moderated by social support.

## Gap Analysis

The existing researches have shown and established the direct influence of social norms, PBC and attitudes, on EIs (Kolvereid, 1996; Van Gelderen et al., 2008; Engle et al., 2010), however, the process through which such predictors influence EIs is still underexplored (Arshad et al., 2016). Bono and McNamara (2011) emphasizes that once the relation among some factors is identified, it becomes necessary to evidently explain the process through which these variables influence each other (Arshad et al., 2016). In the present research, we study a novel path i.e., how an attitude (entrepreneurial commitment, EC) acts as a mediator to elucidate the course by which psychological capital (PC, as a proxy for PBC) develop EIs; how social norms (perceived social support, PSS) strengthens this relationship as a moderator. According to Zhao and colleagues, though researchers have analyzed the influence of psychological capital on performance in offices and organizations, however, they have scantly studied its effect on entrepreneurial intention (EI) or willingness (Zhao et al., 2020).

According to the theory of planned behavior, a person's beliefs influence attitude and modifies their intentions (Ajzen, 1991). Both PC (Bandura, 1997; Bandura et al., 2001) and PSS might affect entrepreneurial attitude and are eventually combined into the entrepreneurial intentions of a person. The research related to social psychology puts forward that several factors affect personnel's attitude and results in intentions (e.g., Park, 2009; Jan et al., 2012). This current research utilizes the framework of the theory of planned behavior in a novel way to underline the share of personal beliefs in generating personal attitude toward behavior (Ajzen and Fishbein, 1975; Bang et al., 2000).

Another research stream in the entrepreneurial intention's literature is role that gender plays in the development of entrepreneurial intentions (Vamvaka et al., 2020). Researchers have shown that career patterns differ across genders and despite the increasing female participation in the enterprise area, the ratio of male entrepreneurs in the industry is almost twice (Shirokova et al., 2016; Zampetakis et al., 2017). This is because males are considered to be predisposed with aggressiveness, independence, courage, and autonomy, which are usually thought to be essential for entrepreneurship (Zhao et al., 2005; Shirokova et al., 2016). Since, it has been established by researches within the framework of the TPB (e.g., Maes et al., 2014; Schlaegel and Koenig, 2014; Shirokova et al., 2016; Zampetakis et al., 2017; Nowiński et al., 2019; Sitaridis and Kitsios, 2019) that gender differential exist with respect to EI, and men exhibit more positive attitudes, PBC, subjective norms, preferences, and intentions for entrepreneurship; the basic goal of this research is to further develop this stream of work by studying the factors (like PC, entrepreneurial commitment, and social support) that can promote EI in females of Pakistan. To our knowledge, the work done in this research is one of the first efforts in the field of entrepreneurial intentions and TPB to analyze the potential direct and indirect influence of PC on EI by the mechanism of entrepreneurial commitment and strengthening effect of social support.

## Literature Review

### Entrepreneurial Intentions (EI)

EI are defined as a person's readiness to perform a particular behavior (Ajzen, 2002). Shinnar et al. (2012) have explained it as an individual's willingness to perform some actions or a desire to follow a particular self-employed career (Bae et al., 2014). EI is dependent on the desire of the individual that motivates him toward the development and implementation of a particular business idea (Lingfei and Li, 2011; Santos et al., 2016) and reflects an individual's passion (Byrne and Fayolle, 2016) to initiate a new venture (Zhang et al., 2014). Opportunity recognition is fundamental to EI (Boyd and Vozikis, 1994) which leads and directs the actions of a person toward the advancement and application of the business idea (Santos et al., 2016). It represents individual's entrepreneurial spirit (Byrne and Fayolle, 2016) or commitment toward initiating and developing a venture (Zhang et al., 2014). This can be seen as the steppingstone in a progressing, long-term entrepreneurship course (Buttar, 2015). Literature has demonstrated that EI largely predicts entrepreneurial behavior (Ajzen, 2002; Kautonen et al., 2015).

### Psychological Capital (PC)

Psychological capital is a personal characteristic of an individual and has been defined as the positive state of psychological development described by self-efficacy, hope, optimism, and resilience (Luthans, 2002). Individual who have strong PC are determinate (Kim-Soon et al., 2013) and can take short term risks (Zhang et al., 2015). PC has been gaining attention in entrepreneurial domain, since it includes mental capabilities that can be measured, advanced, and managed for performance enhancement (Luthans, 2011; Wernsing, 2014). Psychological capital is different from economic, intellectual, and social capital, and consists of an explicit concentration on the psychological state of mind (Sebora and Tantiukoskula, 2014). The positive psychological constructs that defines psychological capital are self-efficacy, optimism, hope, and resilience (Luthans, 2011; Baron et al., 2016).

Self-efficacy signals the beliefs in a person's own capabilities (Bandura, 2000) or the extent of an individual's feelings of being capable to mobilize the enthusiasm, mental resources and courses of action required to effectively complete a particular task (Dissanayake, 2013). Optimism means a general expectation that one will experience a positive outcome in life (Sebora and Tantiukoskula, 2014). It is the extent of likeness or dislike toward a specific career or task (Peprah and Abandoh-sam, 2017). Hope consists of willpower (agency) and way power (pathways) (Wernsing, 2014). Willpower is person's capability to set objectives and enthusiasm to attain those objectives (Sebora and Tantiukoskula, 2014). Way power means an individual's perceived capabilities to create possible routes to achieve desired objectives (Hsu et al., 2014). Lastly, resilience is the degree to which a person is able to rebound back from failure and adjust to fluctuating and worrying life events (Luthans, 2011).

### Entrepreneurial Commitment

Another important aspect of the entrepreneurial process is a person's commitment toward the entrepreneurial attempt and the whole venture start-up process from scratch. Commitment to entrepreneurship mirrors Ajzen and Fishbein's (1980) viewpoint that intention is choice to grab an available opportunity, with a resolute commitment and is characterized by the mindset or approach that the person is focused and determined to create, develop and grow a new business venture (Welsch et al., 2003). Such level of commitment to the entrepreneurial endeavor can also be considered as the passion which is essential for entrepreneurial success (Selz, 1992). It is further characterized by a single-minded concentration to initiate a business and put efforts for its survival and progress, sometimes even at the expense of other worthy and important goals (Welsch et al., 2003).

### Perceived Social Support

Perceived social support has been defined as the mechanism that enables the individual to accept that they are valued and cared (Xiongfei et al., 2019). Likewise, perceived social support explains the perceptions of people that they are a part of society and are valued by others, and if they need any help they will receive it by the people around them (Wills, 1985). It also makes the people confident about their value in the society and make sure that they receive the attentions and support when they need it (Raza et al., 2020). When people feel safe, they feel secure and ultimately make good decisions (Langford et al., 1997). Researchers have elaborated the social support as family, friends, colleagues, neighbors, the community, and their workplaces (Farooq et al., 2018; Kanwal et al., 2019).

From the perspective of TBP literature such support forms the “subjective norms” (Iakovleva et al., 2011; Robledo et al., 2015). It includes beliefs and perception that “reference people” or the close ones would approve their decision to become an entrepreneur. Hence, these referent people set the norms that stipulate how a person would behave (Robledo et al., 2015). Such support enhances an individual's willingness to behave in a certain way, according to the expectation of significant others (Iakovleva et al., 2011). Additionally, if a person believes that entrepreneurial activity is valued in society, because of macro social values and beliefs (Stephan and Uhlaner, 2010; Santos et al., 2016), he or she is more like to opt for it. Therefore, social support in a way serves to legitimatize the choice of entrepreneurial career (Byabashaija and Katono, 2011).

## Research Model

**Figure 1:**
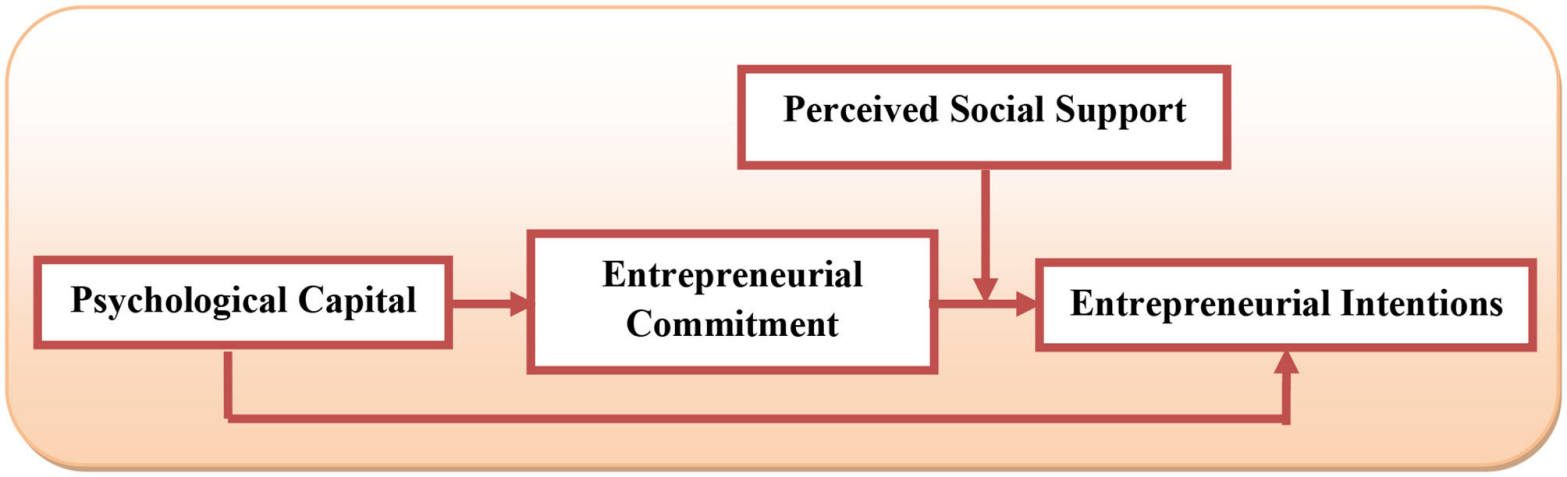
Research framework.

The proposed hypotheses and framework of this study are shown in Figure 1. It is proposed that the psychologically strong females would be more likely to commit to entrepreneurship as a profession. This in turn will result in better intentions toward entrepreneurship. Additionally, if females sense better social support for entrepreneurship, they are more likely to have better entrepreneurial intentions.

## Hypotheses Development

### Entrepreneurial Intentions and Psychological Capital

Psychological attributes play a vital role in entrepreneurial intentions and behavior. A meta-analysis on this variable showed that PC and its constructs are highly related to entrepreneurship (Frese and Gielnik, 2014). Researchers have also argued that there is a positive association between psychological capital and entrepreneurial intention and its dimensions (Contreras et al., 2017b). Psychological capital can boost individual productivity (Luthans et al., 2006; Fenghua et al., 2013). It has been shown earlier that psychological capital is positively related to performance (Yousaf et al., 2015).

Additionally, self-efficacious students have resolute believe in their capability to thrive in entrepreneurial activities (Solesvik, 2017). Also, optimistic students have the ability to identify business prospects where others see anarchy, inconsistency, and confusion (Wernsing, 2014). Hope can help students to take advantage of the opportunities by setting the high standards which they believe they will achieve because they have their eyes on the course to success (Kangarlouei et al., 2012). Resilience in students can aid students in taking risks, and rebound from failures, misfortune, and adversity (Sebora and Tantiukoskula, 2014). Putting it all together, students with high psychological capital would display better EI since they are futuristic (McCann and Vroom, 2015) and tend to take on more challenging work. According to Minniti et al. (2005) half of the working population are women, but they still lag behind male entrepreneurs. Though there are several factors that contributes to women's less involvement in entrepreneurial activities, after having a look at empirical evidence, we have a reason to believe that female entrepreneurs can take advantage from strong psychological capital to build entrepreneurial intention; and hence the following hypothesis is proposed:

**H1: Psychological capital is significantly related to entrepreneurial intentions**.

### Psychological Capital and Entrepreneurial Commitment

A number of prior studies have demonstrated that PC is positively related with commitment (Luthans and Jensen, 2005; Etebarian et al., 2012; Kim et al., 2015). Etebarian et al. (2012) conducted a study in Iran which showed a significant positive association between psychological capital and organizational commitment of employees in a trade organization. Researchers like Luthans and Jensen (2005) have provided empirical evidence for the link between PC, intentions to stay within organization, and commitment toward organizational vision. This research was based on a sample of nurses and suggests that nurses who are psychologically strong might be better in balancing their work and life responsibilities simultaneously and therefore be less inclined to suffer from the exhaustion which increases their chances of leaving the organization or career (Luthans and Jensen, 2005). Moreover, such a positive relationship between PC and EC has also been established by Kim et al. (2015) in a sample of nurses from healthcare industry.

On the basis of discussed literature, we have a reason to believe that female students who are psychologically strong will be better committed to entrepreneurship as a profession. Hence, it is proposed that:

**H2: Psychological capital is significantly positively related to entrepreneurial commitment**.

### Entrepreneurial Commitment and Entrepreneurial Intentions

According to Adam and Fayolle (2015), the concept of commitment must be given due importance in the study of entrepreneurship research since intentions can play a role in the entrepreneurial process. Edelman et al. (2010) has proposed that the intensity of commitment toward cherished goals can explain the decision to take action. In the same vein, Gollwitzer and Brandstatter (1997) have discussed that intentions would not be developed without a strong commitment toward the goal, suggesting that commitment strongly affects intentions toward behavior. Fayolle and Liñán's (2014) application of the theory of commitment to entrepreneurship is worth mentioning in this regard. They define commitment as “devoting one's time, energy, financial, intellectual relational, and emotional resources in a project.”

The three-component model has been widely utilized to assess commitment in the public sector (Liou and Nyhan, 1994), in the organizational context as well as in entrepreneurship research. Sharma and Irving (2005) have applied the concept of commitment to family business. The current study proposes to apply the overall concept of commitment to general entrepreneurship. Just like commitment driven intention aids people in sticking to their objectives even in difficult times (Dholakia and Bagozzi, 2002), likewise in entrepreneurship, the intensity of commitment would have an influence on the entrepreneurial intention-behavior link. In this regard Sahabuddin (2018) has recently shown that confidence level of students to become entrepreneurs is determined by the entrepreneurial commitment through entrepreneurial intentions. This means that EC is directly related to EI. On the basis of this discussion, we construe that:

**H3: Entrepreneurial commitment is significantly positively associated with entrepreneurial intentions**.

### Mediating Role of Entrepreneurial Commitment

TPB has received great attention by the researchers who applied it to the entrepreneurial contexts (Fayolle and Liñán, 2014). Besides all other factors, psychological attributes play an integral role in devising entrepreneurial intentions as also explained in the social cognitive career theory (SCCT) which explain the individual choices for choosing their careers. Another theory by Holland (1997) explains that the interrelationship of environment with a person's cognitive capabilities determines their choices of career and the chances of success (Brown et al., 2006). The strong psychology of a person can motivate him/her to start a new business initiative leading to entrepreneurial intentions. This willingness can translate into commitment of that individual with their career choices. According to Vohora et al. (2004), entrepreneurial commitment should be taken as a distinct dimension of entrepreneurial activities. Vohora et al. (2004) elaborated the concept of entrepreneurial commitment and explained that it as a psychological state of a prospective entrepreneur who focuses on the available opportunities for its exploitation. In a study conducted by Parente and Feola (2013), entrepreneurial commitment played an important role in driving the entrepreneurial intentions. In this context, and based on the discussion provided in H2 and H3, we propose that entrepreneurial commitment is based on psychological factors and plays an important role in the development of entrepreneurial intentions thus developing the hypothesis that:

**H4: Entrepreneurial commitment mediates the positive relation between psychological capital and entrepreneurial intentions**.

### Moderating Role of Perceived Social Support

Individual's positive evaluation of supportive behavior of their significant others make them feel to be more capable of performing the entrepreneurial activities (Kautonen et al., 2013). Scholars have increasingly studied the issues of entrepreneurial intentions during last two decades (e.g., Bird and Jelinek, 1989; Krueger et al., 2000). According to TPB, the individual intentions lead to fruitful actions and their behaviors are shaped by three variables: individual attitude, perceived behavioral control, and social norm (whether or not a specific behavior is acceptable in social context or not) (Kuehn, 2008). Along with the increased emphasis on personality traits, focus has moved toward the impact of social factors which shape entrepreneurial activities (intention and commitment). Such social factors may hinder or promote entrepreneurial activities (Shapero and Sokol, 1982; Begley and Tan, 2001). The role of perceived social support has been explained with entrepreneurial intentions (Baughn et al., 2006; Carr and Sequeira, 2007). The results of the study explain that the people with high social support are more likely to be productive and successful in their careers and tend to take greater part in entrepreneurial activities (Sequeira et al., 2007). Baughn et al. (2006) investigated that social support is a great predictor of entrepreneurial intentions. A study conducted in Pakistan (Sardar et al., 2019) suggests that family plays an important role for women to run their own businesses. On the basis of the importance of social support for female entrepreneurs, we propose that social support would interact with EC to strengthen the indirect relationship between PC and EI, and hence the hypothesis:

**H5: Perceived social support moderates the indirect relation between psychological capital and entrepreneurial intentions through entrepreneurial commitment in such a way that the relationship is stronger when perceived social support is high**.

## Methodology

The study followed a correlational descriptive quantitative research design. Such a research design was aimed at getting a picture of the current thoughts, attitude, and behavioral intentions of female students, as well as to discover relationships among the specified variables and to allow the prediction of future events from present knowledge (Mertens, 2014). According to Mertens (2014), this design provides the benefit of including multiple variables in a single study as compared to other research designs. However, the selection of variables must be based on sound theoretical underpinning rather than on a shotgun approach (Gall et al., 1996).

The philosophical assumptions of a research paradigm denote the set of beliefs about the facts that are under investigation. Four elementary research paradigms were recognized by Guba and Lincoln (2000) in this regard. Because the current study has utilized the existing literature to achieve its research objectives and has utilized theoretical underpinnings of a well-established existing theory i.e., the theory of planned behavior, for the development of hypotheses, therefore, the study is steered by the positivistic research paradigm, with the application of deductive reasoning, as it suits this kind of research (Creswell, 2013).

The study met the philosophical assumptions of positivistic paradigm (Schrag, 1992). The paradigm assumes that there is a procedure for investigating the social world objectively. The positivist researcher is obliged to conduct “good” research which signals “intellectual honesty, the suppression of personal bias, careful collection and accurate reporting of data, and candid admission of the limits of the scientific reliability of empirical studies” (Jennings and Callahan, 1983, p. 6, as cited in Christians, 2005, p. 159). The participants and researchers of the study are assumed to be independent of each other and are therefore thought not to influence each other (Guba and Lincoln, 2000).

From the “Methodological” viewpoint, positivists obtain their methodological approach from natural sciences. But because of the involvement of people in social sciences and difficulty in the application of rigorous scientific methods, positivists usually rely on quantitative tools (Shadish et al., 2002).

Because of the positivism paradigm, the current research began with an observed need to study female's entrepreneurial intentions in Pakistan. Utilizing the existing theory of planned behavior, quantifiable hypotheses were developed, which were analyzed by means of reliable data collected through survey method. Quantitative statistical procedures were applied, consequently resulting in the confirmation of the hypotheses' truthfulness. Positivistic assumptions point to the objective collection of data without the interference of the researcher with the participants. Therefore, non- contrived quantitative research method was adopted by utilizing the survey technique. Research scholars also call it the deductive approach (Sale et al., 2002).

### Data Collection

In quantitative survey designs, the preparation of questionnaire deserves due attention to collect relevant data (Creswell, 2013) in a particular context. We have utilized the Delphi technique to fine tune the asked questions, by seeking the expertise of four specialists (i.e., two experts from the academia, and two female entrepreneurs). During the discussions with these experts, each item was deliberated with respect to its suitability with the aims of this study and target respondents. However, the psychological capital questionnaire (Luthans et al., 2007) was utilized as it is, since it is a well-tested, and reliable instrument. Permission for using this scale was obtained by following relevant protocols. The final questionnaire was made up of two parts in accordance with the time intervals established to collect the responses.

The data for this study was collected from master's in business administration (MBA) students who were females and in final year. The reason for this selection was that MBA students have more knowledge about business related aspects and usually have studied a formal course related to entrepreneurship. Additionally, they are potential entrepreneurs (Fitzsimmons and Douglas, 2011), ready to initiate their careers by acquiring jobs or establishing their own business ventures (Arshad et al., 2016). Because the study is related to female's entrepreneurial intentions, therefore these students were deemed suitable for fulfilling its aims. Convenience sampling method was used because the population list was not available. Data was collected in two waves, for reducing common method bias (Podsakoff et al., 2012). Moreover, Law et al. (2016) proposed that time lagged survey is an effective method to collect data for getting rational results in mediation studies. As English is the official and business language in Pakistani educational institutes and organizations (DeClercq et al., 2017; Jahanzeb and Fatima, 2018) hence the questionnaires were developed and administered in English language.

Because, the population size is unknown for the current study, convenience sampling method was considered to be an appropriate way to choose the participants (Farrokhi and Mahmoudi-Hamidabad, 2012). A well-known and tested formula (i.e., the 5:1 rule; five respondents for every item of the questionnaire) was utilized to calculate the needed sample size set by Kline (2006) and Hair et al. (2007), which yielded required sample size of 305. In behavioral studies, the response rate produced is usually ~60% (Anseel et al., 2010). To get 305 responses, 510 questionnaires were floated. Out of these, 356 questionnaires were filled and returned at T1, meaning that the response rate was 70%. The reason for such a high rate might be because of the personal visits and caution during data collection. At time T2, 356 questionnaires were floated again. This time, 224 responses were received back. Out of these questionnaires, seven had missing values in excess of 15% and were excluded. This missing values exclusion criteria was defined by Hair et al. (2007). The final sample size was 216 (response rate = 43%). The lower response rate is due to the time-lagged study design which was adopted to minimize common method bias and made it difficult to retrieve responses the second time (Jahanzeb and Fatima, 2017).

Because of this low response rate, the adequacy of sample size was calculated by the formula proposed by Tabachnick et al. (2007). According to this formula, *N* > 50 + 8 m, where “*N*” is the minimum sample size and “*m*” is the total variables in the hypothesized model. Consequently, the minimum sample size required for the present study was found to be 82. Furthermore, for a population size of up to 25,000 (at 95% confidence interval, and ± 10% precision), a minimum sample of 100 is adequate (Israel, 1992). Our sample size is also supported by existing studies of similar nature (e.g., Ephrem et al., 2019).

The responses from participants at T1 and T2 were matched by means of a code (birth months and initials) generated through guidance written in the forms. A cover letter explained the respondents about the purpose and significance of this study. Respondents were also informed about the confidentiality of data usage.

### Measures

This study has utilized response options ranging from 1 to 5, unless otherwise mentioned.

### Psychological Capital

We utilized the Psychological Capital Questionnaire (Luthans et al., 2007) for collecting data about female student's psychological capital. The scale is made up of 24 items that measure hope, resilience, optimism, and self-efficacy. A sample item is “I feel confident analyzing a long-term problem to find a solution.” The alpha reliabilities of hope, resilience, optimism, and self-efficacy were 0.77, 0.80, 0.71, and 0.74, respectively.

### Entrepreneurial Commitment

The entrepreneurial commitment was measured by an adopted scale from Vamvaka et al. (2020). The scale consists of 6 items. A sample item is “I will make every effort to start and run my own firm.” The reliability of the scale was 0.79.

### Perceived Social Support

“Instrument on Entrepreneurial Social Support Assessment” (IESSA) was adopted from Sahban et al. (2015) that enabled the researchers to look into the social support system in relation to entrepreneurial desires of students. The scale consists of 25 items with 15 items for family support and 10 for peer support. A sample item was “My family supports me in making career decisions.” The alpha reliability of the scale was 0.81.

### Entrepreneurial Intentions

For measuring the entrepreneurial intentions among female students, we used Entrepreneurial intention questionnaire (Liñán and Chen, 2009). It has six items, with a sample item: “I am ready to do anything to be an entrepreneur.” The Cronbach alpha reliability for the scale was 0.89.

## Data Analysis

### Results

Table 1 shows the descriptive statistics and intercorrelation among the variables, whereas the results regarding regression are displayed in Tables 2, 3. Before moving on to regression analysis, the variance inflation factor values were checked for each model of regression coefficients. All the values were found to be below 10, showing that the data was free from the problem of multicollinearity (Aiken et al., 1991). Moreover, the numbers in Table 1 show a moderate correlation among the studied variables which are in line with the standards (Cohen et al., 2014).

**Table 1:** Correlation analysis.

**Constructs**	**Mean**	**SD**	**MS**	**A**	**PC**	**EC**	**SS**	**EI**
Marital Status (MS)	1.1	0.51	1					
Age (A)	2.45	1.11	0.46^**^	1				
Psychological Capital (PC)	2.04	0.77	0.19^**^	−0.12	1			
Ent. Commitment (EC)	2.4	0.81	0.03	0.01	0.36^**^	1		
Social Support (SS)	2.66	0.70	−0.13	0.03	0.15^*^	0.4^**^	1	
Ent. Intentions (EI)	1.9	0.80	0.10	−0.08	0.63^**^	0.52^**^	0.30^**^	1

*N = 216, age and marital status are control variable*,

* *ρ < 0.05*,

** *ρ < 0.01. Ent, Entrepreneurial*.

**Table 2:** Mediating effect of EC between PC and EI using process macro.

**Predictor**	**Model 1 (path c)**			**Model 2 (path a)**			**Model 3 (path b and c)**		
	**EI**			**EC**			**EI**		
	**β**	***t***	**CI**	**β**	***T***	**CI**	**β**	***t***	**CI**
PC	0.64^*^	11.61	0.53; 0.75	0.34^*^	5.7	0.25; 0.52	0.52^*^	9.3	0.4; 0.6
EC							0.33^*^	6.2	0.23; 0.44
*R^2^*	0.39^*^			0.14^*^			0.49^*^		
F	134.9			33.4			99.8		

* *ρ < 0.001, 95% confidence interval, 5,000 bootstrap samples*.

**Table 3:** Moderated mediation effect of PSS using process macro.

**Predictor**	**Outcome variable**			
	**EI**			
	**β**	**T**	**CI**	***R^**2**^***
PC	0.52^*^	9.7	0.4;0.6	
EC	−1.2	−0.65	−0.5;0.25	
PSS	−0.25	−1.4	−0.6;0.09	
EC^*^PSS	0.15^**^	2.3	0.02;0.28	0.51^*^ (*F* = 54.05)

* *ρ < 0.05*,

** *ρ < 0.01, 95% confidence interval, 5,000 bootstrap samples*.

In order to test hypotheses 1, 2, and 3, hierarchical regression test was applied. The results showed support for the first hypothesis which predicted a positive relationship between PC and EI. The figures (β = 0.64, *t* = 11.61, *p* < 0.001) in Table 2, Model 1 indicates that as the psychological capital increases, the intentions of pursuing entrepreneurship as a profession in female students also increase.

Hypothesis 2 had suggested that females who have abundant psychological resources will be more committed to entrepreneurship. In support of this prediction, Model 2 in Table 2 demonstrates a positive association between PC and EC (β = 0.34, *t* = 5.7, *p* < 0.001). Because females with more PC are more optimistic, hopeful, resilient and confident, there are more chances that they will be more committed toward adopting entrepreneurship as a career choice. Additionally, Hypothesis 3 predicted that the more are the females committed to entrepreneurship, the higher will be the level of EI in them, as shown by the positive relation between EC and EI in Model 3 of Table 2 (β = 0.33, *t* = 6.2 *p* < 0.001).

Hypothesis 4 of this study proposed that EC mediates the relation between PC and EI. For testing the mediating relationship, we utilized the Preacher and Hayes's (2004) technique and executed it at 5,000 bootstrapping, by using the Process macro (Hayes, 2017). This technique produces confidence intervals (CI) for the indirect effects. Therefore, this technique reduces the potential statistical power problems that may arise from non-normal or asymmetric sampling distributions (MacKinnon et al., 2004). The CI for the indirect effect of PC on EI *via* EC did not include 0 (CI = 0.07; 0.19) and hence supported of the presence of mediation.

Regression results presented in Table 2 contain three models to explain the statistics extracted for mediation analysis. Model 1 shows that PC significantly positively predicted EI and 39% variation found in EI was due to PC (*R*^2^ = 0.39, *F* = 134.9, *p* < 0.001). Next, Model 2 demonstrates a positive relation between PC and EC with *R*^2^ value of 0.14 (*F* = 33.4, *p* < 0.001). In model 3, after controlling for PC, EC positively affected EI with *R*^2^ value of.49. Finally, biased corrected percentile bootstrap method using model 4 of PROCESS macro by Hayes (2017) indicated that indirect path of PC on EI through EC was satisfied with these values, *b* = 0.13, SE = 0.03, 95% (0.07, 0.19). Therefore, hypothesis 4 was accepted, supporting the mediation effect of EC between PC and EI.

The last hypothesis of the study was to examine the moderating role of PSS in the indirect relation between PC and EI *via* EC. To examine this moderated mediation relationship in Hypothesis 5, we used Preacher et al. (2007) procedure and Hayes's (2017) Process macro i.e., Model 14. This process produces CI for the conditional indirect effects (MacKinnon et al., 2004). The results from the analysis with 10,000 random samples and replacement from the full sample, showed that 95% bootstrap confidence intervals for the conditional indirect effect of PC on EI at the low level (−1 SD) of the moderator POR did not contain zero at low (*b* = 0.06, CI = 0.01; 0.13), or high (*b* = 0.15, CI = 0.04; 0.23) values of PSS. However, the effect size was greater when the values of PSS were higher as compared to when they were lower, showing that the presence of higher social support further strengthens the indirect relation between PC and EI through EC. Moreover, the index (Hayes, 2017) of moderated mediation (*i* = 0.59) and its corresponding confidence interval did not include zero (CI = 0.007; 0.11), showing that PSS further reinforces the indirect influence of psychological capital on entrepreneurial intentions, through entrepreneurial commitment, hence supporting the last Hypothesis and the study's overall framework. Table 3 shows that the interaction term EC × PSS was found to be significant (β = 0.15, *t* = 2.3, *p* < 0.001) with a ΔR^2^ =0.012, (*F* = 5.25, *p* < 0.05); demonstrating that the positive effect of EC on EI was further strengthened by higher levels of perceived social support for entrepreneurship.

**Figure 2:**
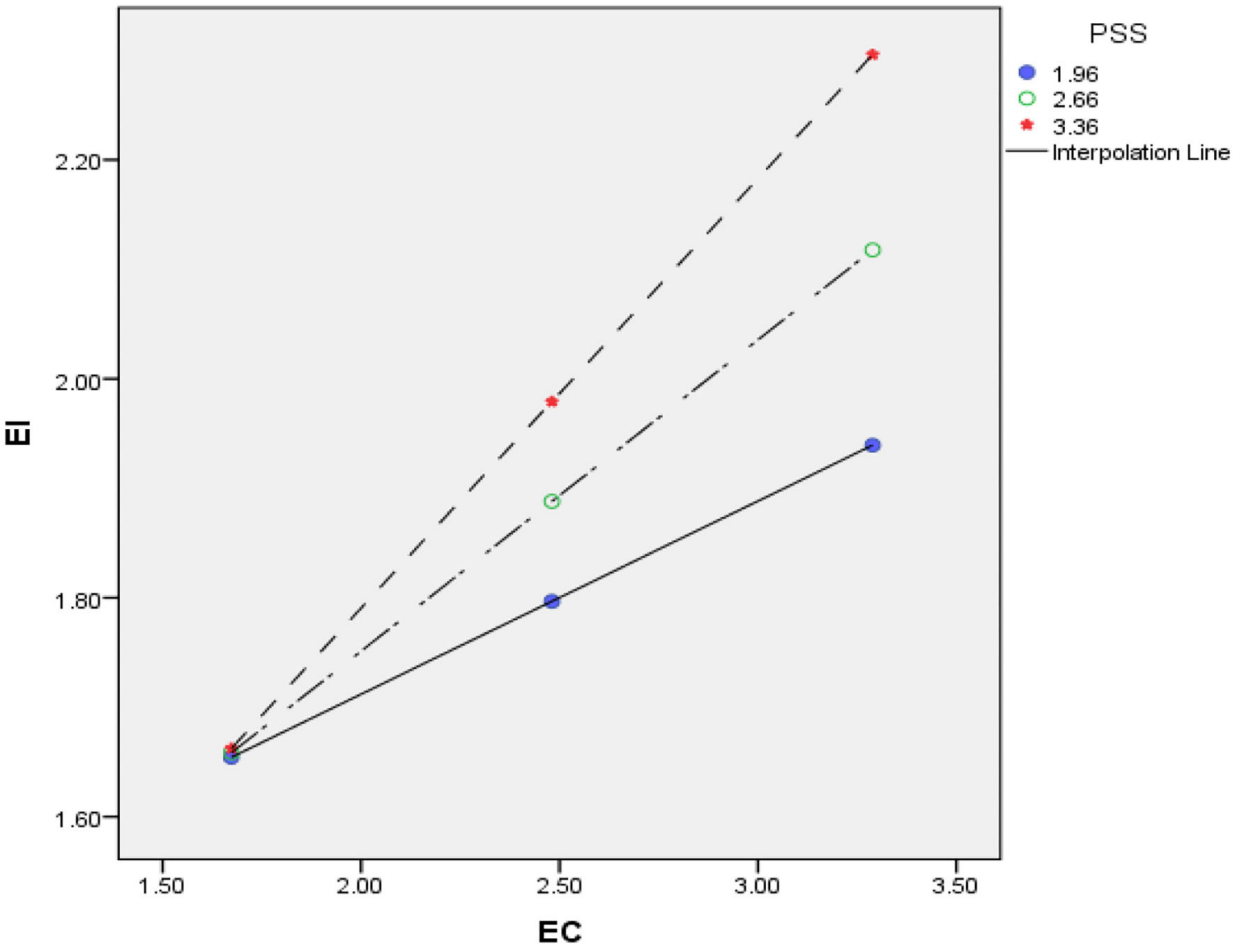
Moderating role of PSS in the relation between EC and EI.

A simple slope test (Aiken et al., 1991) showed that at different values of PSS, the effect of EC on EI were significantly different; for example at low levels of PSS, the relation between EC and EI was found to be less strong however at high levels of PSS, the relation was more stronger. The significance of the interaction is further validated by plotting the values in a graph with moderating variable at +1 SD and −1 SD. Figure 2 shows the effect of EC on EI at high, medium and low levels of PSS. The effect of EC on EI is further strengthened at high levels of perceived positive social support.

## Conclusion and Discussion

The results from this study have further extended the existing knowledge base (e.g., Arshad et al., 2016; Shirokova et al., 2016; Zampetakis et al., 2017) and concluded that psychological capital, entrepreneurial commitment, and social support collaboratively and interactively affected the entrepreneurial intentions of female MBA students in Pakistan. Entrepreneurial commitment plays a mediating role between the relation of psychological capital and entrepreneurial intentions. Next, perceived social support was found to be a moderator in this indirect relation in such a way that high perceptions of social support further strengthened the said association. These findings have found support in the theory of planned behavior in that entrepreneurial intentions are influenced by attitudinal, social, and psychological aspects. It is therefore recommended that policy makers in Pakistan inculcate their female students with psychological capital. The entrepreneurial knowledge and benefits through on-campus activities intended to build female's entrepreneurial intentions must be promoted, so that this untapped resource can be used to further the economic benefits for the country.

Additionally, developing and preparing more entrepreneurs is one potential way of reducing unemployment in Pakistan. Due to limited employment opportunities, the number of unemployed workers is increasing. Especially as the number of educated lots is increasing, unemployment despite having adequate education posits real threat to people as well the society. Furthermore, it is a recognized truth that females come across more problems in initiating and advancing ventures (Heilbrunn, 2004; Marlow and Carter, 2004; Thébaud, 2015). Even in the presence of beneficial participation of women in economic steadiness (Kelley et al., 2013; Klasen and Pieters, 2015; Westhead and Zolesvik, 2016; Ng and Sears, 2017), a gender differential disparity still exists between entrepreneurs (Shiri et al., 2017). Women show lesser intentions of becoming entrepreneurs (Shane, 2008; Kelley et al., 2013), as they see the profession as male central and a masculine choice (Henry et al., 2016). Some scholars think that female entrepreneurs are generally at a disadvantage as compared to men (De Bruin et al., 2006). The work-related difficulties and complexities are even more noticeable for females in the developing countries (Madsen and Scribner, 2017; Sarwar and Imran, 2019), where they have inadequate access to technology and education. In these nations, female involvement in the work force is usually more conventional, and prospects of economic progress are less (Sullivan et al., 1997). Earlier researchers (Altinay et al., 2012; Barba-Sánchez and Atienza-Sahuquillo, 2018) have shown that EIs can boost the initiation of new ventures. We have protracted this knowledge by studying the antecedents and strengtheners of EI due to which the unemployment problem in Pakistan might be catered to some extent and offered advanced comprehension of the process underlying this phenomenon especially as females represent an untapped pool of resources.

Moreover, earlier, some scholars (e.g., Arshad et al., 2016) showed that in Pakistan, entrepreneurial self-efficacy is more imperative for men in defining the attitudes toward EI as compared to women. We have shown that not only self-efficacy, but other aspects of psychological capital including hope, resilience, and optimism plays a vital part to advance EI in women of Pakistan. But because men usually have more control on socio-economic resources in South Asian (Agarwal and Bina, 1994) settings, so it becomes important for women to get aid and support from society for furthering their EIs.

## Theoretical Contributions

Our study makes several additional contributions. We have introduced the mediating effect of entrepreneurial commitment in the association between psychological capital and EIs. Some scholars have suggested that a person's belief system (in the form of hope, optimism, resilience, and self-efficacy) influence the attitude toward behavior, and consequently such attitude regulate their intentions (Bang et al., 2000; Lee and Peterson, 2000; Chuttur, 2009). TPB also endorses entrepreneurial commitment as mediator amid a person's beliefs and EI (Fishbein and Ajzen, 1975; Bang et al., 2000). Though extant research has investigated direct influence of attitude, social norms, and PBC toward a person's entrepreneurial intentions (Kolvereid, 1996; Van Gelderen et al., 2008; Engle et al., 2010), our study has shown that psychological capital has the ability to affect entrepreneurial intentions by the mediation of entrepreneurial commitment. Hence our study extends previous researches that confirmed a direct link of attitudes and PBC on entrepreneurial intentions (Chen et al., 1998; Krueger et al., 2000).

Our findings showed an indirect influence of PC on entrepreneurial intentions through entrepreneurial commitment. We reaffirm the findings of Tsai et al. (2016) who demonstrated an indirect link of self-efficacy with entrepreneurial intentions through entrepreneurial attitude but ignored to take into account other dimensions of psychological capital including hope, optimism, and resilience. Additionally, earlier, Kolvereid (1996) showed a direct relation between social norms and EIs. We on the other hand have shown that social support plays a moderating role between the indirect link of PC and EI *via* EC. The said indirect association was further strengthened in the presence of social support. We therefore have described the moderated mechanism by which psychological capital and entrepreneurial commitment effect entrepreneurial intentions *via* commitment and thus offer additional academic thoughts on the chain of effects of psychological capital on EIs *via* EC.

Our findings also provide evidence of direct positive effect of psychological capital and attitudes on EIs. These results reaffirm Autio et al.'s (2001) findings of a positive association among attitude and entrepreneurial intentions in the USA background. However, Siu and Lo (2013) found an insignificant association among attitude and entrepreneurial intentions in China. Hence, it seems that the association among attitude and intentions is varying and might differ by contextual settings. We invite future researchers to explore further other moderating and mediating variables that might alter the association between attitude and EIs across various cultures. The findings of this research provide evidence that TPB (Ajzen, 1991) offers ideal avenue for comprehending a person's entrepreneurial intentions in developing nations and affirms that entrepreneurial attitude, psychological capital, and social support are the antecedents of a person's EIs (Kolvereid, 1996; Engle et al., 2010).

Because entrepreneurship enhances a country's advancement and socio-economic growth (Romer, 1994), it has become vital for developing nations. The research about EIs in developing nations might aid in the better comprehension of reasons about lesser EI. Since female's inclination toward entrepreneurship is usually low, in comparison to men (Jonathan and Mark, 2011; Santos et al., 2016), it is essential to find the issues that might boost entrepreneurial intentions in women. We have carried out this study in South Asian context, specifically Pakistan (a patriarchal society) where the economic and cultural aspects are in sharp contrast to each other and women's career outlooks are not as promising as those in developed nations. To study the female's entrepreneurial intentions in South Asia, we therefore used the TPB (Ajzen, 1991) and showed that females with stronger psychology, with higher levels of hope, optimism, resilience, and self-efficacy are better able to develop commitment for entrepreneurship which later translates into EI. Additionally, we also showed that social support plays a significant role in enhancing their attitude toward entrepreneurial intentions. This means that when women perceive that the society backs up their entrepreneurial commitment, they feel encouraged to start their own venture. This might be because women of conservative cultures like Pakistan are more motivated to follow the social norms (Arshad et al., 2016). It can be due to several factors. One such reason may be that most of the women here are given social and financial support from their close ones (Agarwal and Bina, 1994, Sarwar and Imran, 2019). Therefore, it is quite possible that they may not be in a place to make important decisions on their own like initiating a venture, without finding preceding support from their close relatives.

In the GEM report, Qureshi and Sarfraz (2012) suggested that entrepreneurship is largely thought to be a male field in Pakistan, and that there is less involvement of females in business side. Hence it appears that women enjoy very little social support for starting new business venture (Arshad et al., 2016). Our study has revealed that once the females of Pakistan perceive that they have support of the society, they develop comparatively stronger positive attitude toward entrepreneurial intentions. Earlier some scholars (e.g., Díaz-García and Jiménez-Moreno, 2010) have studied the moderating role of gender between subjective norms and EI such that the effect of subjective norms on entrepreneurial intentions is more for females than for males. Our research extended their results by showing that social support aids and strengthens the link between attitudes and EIs. Furthermore, our study presents an alternate perspective from a developing nation's cultural and socio-economic context in contrast to findings from western countries (e.g., Díaz-García and Jiménez-Moreno, 2010).

## Practical Implications

The findings of current research have several important implications for policy makers in developing and especially patriarchal countries. As per our findings psychological capital is an important predictor of entrepreneurial commitment and EI in females. Leaders should make strategies to increase entrepreneurial activity in the country by inculcating more psychological capital in female students, by teaching them psychology related courses that might boost their confidence, hopes, resilience, and optimism. Some earlier studies have shown that women possess less self-efficacy (Wilson et al., 2007; Mueller and Dato-On, 2008). But because women also make half of the world population, they are capable of positively contributing toward the economic development. Hence, it would be fruitful if they can be strengthened mentally. Entrepreneurial learning should be coordinated with the development of specific vocational skills so that female graduates of business management—besides being competent to work in industry—are also trained to create new business opportunities leveraging their vocational expertise.

Additionally, role modeling (BarNir et al., 2011), training and education (Schunk, 1995) might improve the confidence of the female students. Certain programs might be introduced at college and university levels whereby female students are motivated by success stories of female entrepreneurs and role models. Talks might be arranged by these female role models that might inculcate hope, optimism, resilience, and self-efficacy in students who can later on become entrepreneurs. Training and knowledge programs related to entrepreneurship have the capacity to advance entrepreneurial efficacy (Schunk, 1995). Additionally, because social support also played a part in strengthening the EI of females, therefore leadership must try and encourage entrepreneurial culture among general masses and educate everyone about the benefits of female's entrepreneurship. Government should offer packages for females to start their own ventures for increasing peer pressure on women to improve attitudes and intentions related to entrepreneurship.

Our findings are in line with Patel and Thatcher's (2014) findings that individual attributes play a significant role in entering and persisting in entrepreneurship. We therefore recommended that organizations involved in entrepreneurial supports should make efforts for boost PC in entrepreneurial support programs, because most of the aspects of PC can be learnt. It will also be fruitful if governments could include provisions regarding the issues highlighted by us in their entrepreneurship policies. The influence of psychological capital on entrepreneurial intentions, directly and indirectly, and the moderation of social support has implications for career counseling, guidance, and training. Entrepreneurship is an exciting profession that involves challenging situations and necessitates individuals to make vital decisions on day to day basis. The results of this study suggest that career counselors and trainers must pay attention to the improvement of entrepreneurs' psychological capital, including the aspects of hope, resilience, optimism, and self-efficacy. Similarly, female students must be encouraged to seek opportunities to strengthen their positive psychological characteristics. Psychologists might organize seminars and exercises specifically for female micro- and prospecting entrepreneurs meant to improve their psychological capital.

## Limitations and Future Directions

Even with several important contributions, the study is not without limitations. The first one is related to contextual limitations. Because the results are based on data collected from Pakistani universities, the generalizability might be limited. Future scholars should validate the results by more studies in other contexts since entrepreneurial activities differ in various settings (Fernández-Serrano and Romero, 2014). Though we utilized a multidimensional construct (Tsai et al., 2016) for PBC in the form of psychological capital, future researchers are invited to test such other constructs. Our findings have shown that PBC can influence EIs *via* attitude, however some scholars have found insignificant influence of attitude toward entrepreneurship on EIs (Siu and Lo, 2013). Such inconsistent results point toward the need for more moderating variables that might enlighten the circumstances in which entrepreneurial attitude more strongly influences EIs.

Additionally, because data was collected from female university students, it would be useful that data be collected from other occupations in future studies. As this study utilized the theory of planned behavior and did not consider other variables associated with entrepreneurial intentions, it did not offer a whole depiction of EIs. When comparing the findings of this study with others, it is noted that association of predictors from the TPB perspective with respect to EIs differs across various countries. Therefore, future research must consider cultural norms while studying EIs.

## Data Availability Statement

The raw data supporting the conclusions of this article will be made available by the authors, without undue reservation.

## Ethics Statement

The studies involving human participants were reviewed and approved by Comsats University Islamabad. The patients/participants provided their written informed consent to participate in this study.

## Author Contributions

AS designed the study, wrote first draft, and collected and analyzed the data. QA supported in data collection and in hypotheses development section. NR helped in data collected and write up. All authors revised and agreed to final submitted version.

## Conflict of Interest

The authors declare that the research was conducted in the absence of any commercial or financial relationships that could be construed as a potential conflict of interest.
